# Genome Sequences of Two Pathogenic *Streptococcus agalactiae* Isolates from the One-Humped Camel *Camelus dromedarius*

**DOI:** 10.1128/genomeA.00515-13

**Published:** 2013-07-18

**Authors:** Saima Zubair, Etienne P. de Villiers, Mario Younan, Göran Andersson, Herve Tettelin, David R. Riley, Joerg Jores, Erik Bongcam-Rudloff, Richard P. Bishop

**Affiliations:** Department of Animal Breeding and Genetics, SLU Global Bioinformatics Centre, Swedish University of Agricultural Sciences, Uppsala, Swedena; International Livestock Research Institute, Nairobi, Kenyab; Institute for Genome Sciences, University of Maryland, School of Medicine, Baltimore, Maryland, USAc; Tierärzte ohne Grenzen/Vétérinaires sans Frontières Germany, Nairobi, Kenyad

## Abstract

*Streptococcus agalactiae* causes a range of clinical syndromes in camels (*Camelus dromedarius*). We report the genome sequences of two *S. agalactiae* isolates that induce abscesses in Kenyan camels. These genomes provide novel data on the composition of the *S. agalactiae* “pan genome” and reveal the presence of multiple genomic islands.

## GENOME ANNOUNCEMENT

*Streptococcus agalactiae*, also known as group B *Streptococcus* (GBS), is an emerging human pathogen, mainly in neonates (1). *S. agalactiae* infection occurs frequently in camels (*Camelus dromedarius*) and can result in mastitis (2), abscesses, and respiratory tract infections (3). However, the molecular basis of tissue tropism and multiple clinical syndromes is unknown. We have determined and annotated the genome sequences of two different *S. agalactiae* isolates, ILRI005 and ILRI112, associated with abscesses from Kenyan *C. dromedarius*. These pathogenic isolates are genetically distinct according to multilocus sequence typing (A. Fischer, A. M. Liljander, H. Kaspar, C. Muriuki, H. Fuxelius, E. Bongcam-Rudloff, E. deVilliers, C. A. Huber, J. Frey, C. A. Daubenberger, R. Bishop, M. Younan, and J. Jores, submitted for publication).

Sequencing of the *S. agalactiae* isolate ILRI005 was performed using an Illumina genome analyzer (GA) IIx with paired-end-read libraries with a mean library insert size of 210 bp and an average read length of 100 bp. For mapping and *de novo* assembly of 20,687,942 quality reads, we used MIRA v 3.0.0 (4). For mapping we used the genome sequence of the bovine *S. agalactiae* isolate 09mas018883:HF952104 as a reference template (4a). Contigs generated by the *de novo* assembly were ordered using the reference genome, and the consensus genome sequences were aligned using Mauve (5). Genome finishing employed a combination of comparative assembly plus PCR amplification and Sanger sequencing of gaps and GapFiller (6) and Velvet (7) software. A total of 20,189,204 reads (97.56%) were aligned to the reference genome, with an average coverage of 936×. The ILRI112 isolate was sequenced using Ion Torrent technology with a single end-read library with an average read length of 200 bp. Mira v 3.4.1.1 was used to assemble 3,123,413 quality reads. The combination of mapping and *de novo* assembly approaches generated a complete genome sequence with 96% total read alignment and 224× average coverage. Annotation and analysis of the genomes were performed using Basys (8) and RAST (9), Artemis, the Artemis Comparison Tool (10), and Sybil (11).

ILRI005 comprised 2,109,759 bp and ILRI112 2,029,198 bp, with 35.34% and 35.43% GC content, respectively. Identity between homologous regions of the two camel isolate genomes was 99.885% based on JSpeciesv1.2.1 analysis (12). A total of 2,134 open reading frames (ORFs) were predicted in the genome of ILRI005, compared to 2,048 in ILRI112. ILRI005 contained 1,846 genes shared with other *S. agalactiae* strains and 288 unique genes, whereas the ILRI112 genome contained 1,911 shared genes and 137 additional ORFs. Approximately 70% of the predicted ORFs had a putative assigned function.

Genomic islands were predicted using Island Viewer (13). Isolate ILRI005 contained 6 putative genomic islands incorporating 76 predicted genes, whereas ILRI112 contained 7 genomic islands with 117 genes. In contrast to other GBS strains, ILRI005 had an insertion in the region encoding the capsular polysaccharide (cps) (14) of approximately 4,000 bp carrying 8 predicted ORFs, including the *cpsG* and *cpsH* genes.

These genomes provide additional data on the composition of the *S. agalactiae* “pan genome.” Their availability will enable the identification of genes encoding candidate virulence and tissue tropism determinants and the development of specific markers for camel isolates within the type B *Streptococcus* complex.

### Nucleotide sequence accession numbers.

The ILRI005 and ILRI112 genomes have been deposited in ENA under accession numbers HF952105 and HF952106.
